# The Epidemiological Society of London

**Published:** 1855

**Authors:** 


### THE EPIDEMIOLOGICAL SOCIETY OF LONDON.

1. The Transactions of the Epidemiological Society of London, now for the first time published officially, will form one of the most interesting and valuable departments of THE JOURNAL OF PUBLIC HEALTH.

2. The Society was founded a few years ago on the suggestion of Mr. Tucker, now one of its honorary and indefatigable secretaries; and its objects have been and are of a character second to none in interest and national importance.

3. The labours of the Society are directed to one special end—the investigation of epidemic or spreading diseases. It inquires into the causes of these diseases, their prevalence, their relationship to each other, their removal, and their prevention.

4. From the time when the Society was first established to the present, it has been felt by the council and members, that, in conducting their extensive inquiries, much assistance might be obtained from other classes of the community than the medical profession. The membership of the Society is, therefore, thrown freely open to the public; and its honorary distinctions have been conferred on numerous eminent politicians and public men. We hope that, as the objects and constitution of the Society become more widely known, large numbers of general and scientific scholars will give their invaluable assistance, as subscribers and working members. It is by such sympathy and co-operation that the ultimate benefits of this institution will be most speedily and fully developed.

5. The Society has already performed many labours of great public utility. By the exertions of one of its committees, it has been enabled to present to the Government the most comprehensive report on Small Pox and Vaccination that has ever been published; and, within these last few weeks, a most elaborate, practical, and suggestive memoir on the subject of Vaccination has been presented to the Board of Health.

6. Taking a very wide view of the subject to which it is specially devoted, the Society has of late been inquiring into the epidemic diseases of inferior animals; on which question the council have now a vast store of information, waiting only for arrangement and publication.

7. We mention these labours of the Epidemiological Society as a kind of prelude to two or three observations, which it is very painful to make; viz., that as yet the exertions of the Society have been mainly carried on by a few industrious and enthusiastic individuals, most of whom are of the medical profession; that these exertions have scarcely been recognized or seconded by the public at large; and that much of the usefulness and efficiency of the Society is held in check by the mere want of means for carrying on its expensive investigations.

8. This unfortunate result does not, however, as we believe, arise from any want of sympathy on the part of the public with works so useful as those which the Society endeavours to perform; but rather from the fact, that the public as yet know but little of the Society, its labours, desires, and deserts.

9. In the course of the present session, in its anxiety to carry out certain inquiries, the council of the Society petitioned the Lords of Her Majesty's Treasury for a grant of money to enable them to proceed. This request, from motives of policy, in themselves prudent and sound, though in the present case seemingly harsh, was refused. The Lords of the Treasury felt it impossible to make an exception to the general rule of affording no grants to private and unchartered associations.

10. This application to the Government having failed, it has been suggested to the council by Sir Charles Mansfield Clarke, that the Society should appeal for support to the public at large; and the worthy baronet has promised to lead the way by offering himself a handsome pecuniary donation.

11. Were the suggestions of Sir Charles Clarke to be acted upon by the Society, its results cannot be doubted; but in our opinion such a step is unnecessary. The English people want no solicitations to assist in forwarding works which are at once widely useful, honourable, and lasting. Let it but become generally known that a Society, full of mental vigour, and anxious ever to add to our national greatness, is struggling in its efforts from the simple want of a little Californian treasure, and the golden shower will not hang long in the clouds.

12. To the usefulness of the labour of such a learned society as the Epidemiological, there is no end. The accomplishment of one of the smallest of its objects might repay with interest, and compound interest, a Rothschild's wealth for ages to come, to say no word of the happiness it might also procure. Who can place any adequate value on Jenner's simple but great discovery? Who can say that many such discoveries, which it is the strict business of the Epidemiological Society to disclose, are not in store, or even close at hand?

